# Glaucoma and Systemic Disease

**DOI:** 10.3390/life13041018

**Published:** 2023-04-15

**Authors:** Eugene Hsu, Manishi Desai

**Affiliations:** Department of Ophthalmology, Boston University School of Medicine, 85 East Concord Street, 8th Floor, Boston, MA 02118, USA; eugene.hsu@bmc.org

**Keywords:** ophthalmology, glaucoma, open-angle glaucoma, closed-angle glaucoma, systemic disease, acute angle closure glaucoma

## Abstract

Glaucoma is the leading cause of irreversible blindness in the world. Due to its potential to cause permanent vision loss, it is important to understand how systemic conditions and their respective treatments can be associated with or increase the risk for developing glaucoma. In this review, we examined the literature for up-to-date discussions and provided commentary on glaucoma, its pathophysiology, and associated risk factors. We discuss systemic diseases and the impact, risk, and mechanism for developing glaucoma, including pharmacologically induced glaucoma; inflammatory and auto-immune conditions; infectious, dermatologic, cardiovascular, pulmonary, renal, urologic, neurologic, psychiatric and systemic malignancies: intraocular tumors; as well as pediatric, and genetic conditions. The goal of our discussion of systemic conditions including their commonality, mechanisms, treatments, and associations with developing glaucoma is to emphasize the importance of ocular examinations and follow-up with the multidisciplinary teams involved in the care of each patient to prevent unnecessary vision-loss.

## 1. Glaucoma Introduction and Definition

### 1.1. Introduction and Epidemiology of Glaucoma

Glaucoma is the leading cause of irreversible blindness in the world [1]. In 2010, 60.5 million people were estimated to have glaucoma. In 2020, this number increased to 79.6 million [2]. Glaucoma is more prevalent in persons of African and Hispanic descent [3,4], and patients with glaucoma can be asymptomatic for many years, making it likely that these measurements underestimate the true prevalence of the disease.

Glaucoma is separated into two categories: open-angle and angle-closure. In the United States, more than 80% of patients have open-angle glaucoma, but closed-angle glaucoma contribute a significantly higher proportion of cases of severe vision loss. It is estimated that 68.56 million people worldwide have primary open-angle glaucoma [2] and 17 million people worldwide have primary closed-angle glaucoma [2]. Both categories of glaucoma can be acquired as primary or secondary diseases. Secondary causes of glaucoma be medications such as corticosteroids, systemic inflammation, intraocular and systemic tumors, or other predisposing conditions such as diabetes, hypertension, or genetic syndromes. Because of the permanent and lasting effects, it is important to understand how systemic conditions and their treatment can be associated with or lead to glaucoma. Before embarking on this discussion, it is useful to have a deeper understanding of glaucoma and its pathophysiology.

**Figure 1:**
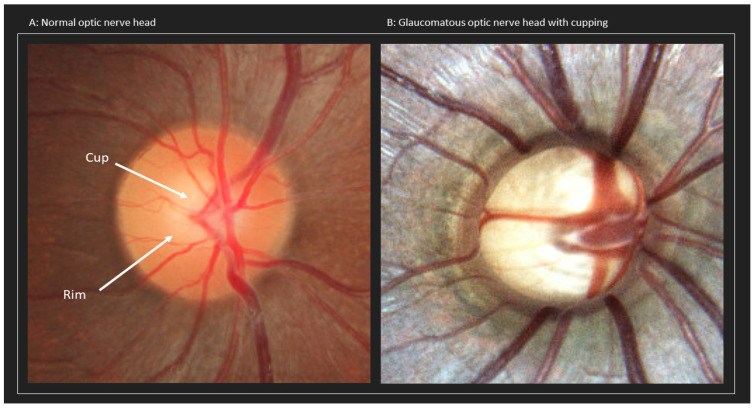
(**A**) Normal optic nerve. (**B**) Glaucomatous optic nerve with cupping.

Glaucoma is a degenerative optic neuropathy stemming from damage and progressive gradual loss of retinal ganglion cell (RGC) axons. The cell bodies of the RGCs are in the inner retina, and their axons comprise the optic nerve. They synapse in the lateral geniculate body. The gradual degeneration of RGCs and RGC axons of the optic nerve leads to the characteristic cupping of the optic nerve seen in ophthalmoscopy (see Figure 1B) and subsequent visual field defects. Treatment can slow the progression of glaucoma, but there is no curative treatment. It is often, but not necessarily, associated with increased intraocular pressure (IOP).

### 1.2. What Defines Intraocular Pressure?

**Figure 2:**
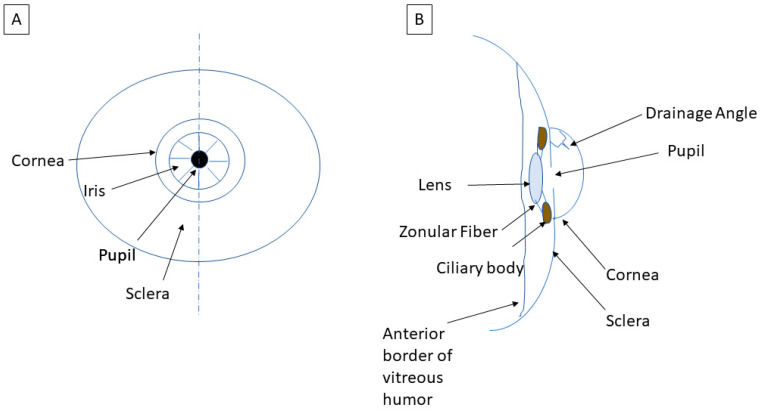
Sketch of the anatomy of the eye. (**A**) Anterior view of the cornea, iris, pupil and sclera and (**B**) cross-sectional view. (Schematic by M.D.).

**Figure 3:**
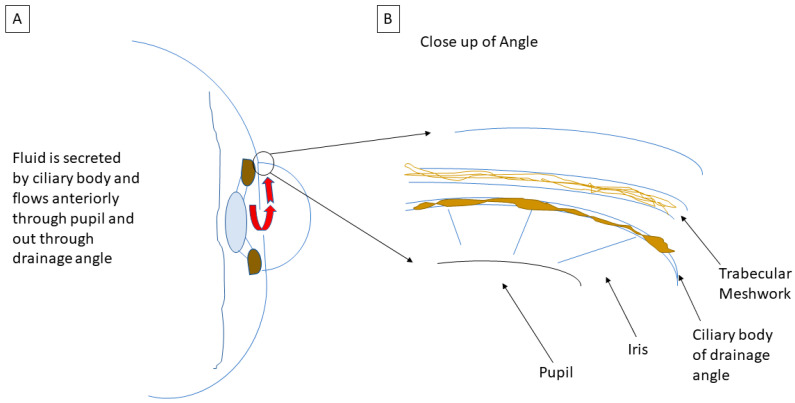
Sketch of (**A**) the flow of aqueous humor (red arrows) secreted by the ciliary body from the posterior chamber into the anterior chamber through the pupil and exiting through the drainage angle. (**B**) Close up of the drainage angle and its relevant anatomy. (Schematic by M.D.).

The IOP, in simplified terms, is the balance between the production and outflow of aqueous humor. Aqueous humor is the intraocular fluid that is produced to nourish ocular structures, and it is produced by the ciliary body, a tissue structure located posterior to the iris, which also suspends the intraocular lens (see Figure 2B). The secreted aqueous humor flows from behind the iris into the anterior chamber through the pupil and out of the eye via the drainage angle (see Figure 3A).

The aqueous humor drains primarily through the trabecular meshwork, with a lesser, but unknown amount, exiting via the uveoscleral outflow pathway. Whether this trabecular meshwork is “open”, i.e., physically unobstructed, helps to define the type of glaucoma as open-angle or closed-angle.

### 1.3. Mechanism of Open-Angle Glaucoma

Open-angle glaucoma occurs when there is increased resistance to the outflow of aqueous humor within the trabecular meshwork itself [5], but without visible obstruction to the drainage angle. In open-angle glaucoma, there is dysfunction in the trabecular meshwork or subsequent area of drainage called Schlemm’s canal, leading to elevated IOP [5]. There is also a subset of open-angle glaucoma patients that do not have elevated pressures, termed normal-tension glaucoma (NTG). While the exact pathophysiology is unknown, the presence of glaucomatous change in the absence of elevated IOP suggests that other mechanisms may play a role in the development of glaucoma.

### 1.4. Mechanisms of Closed-Angle Glaucoma

**Figure 4:**
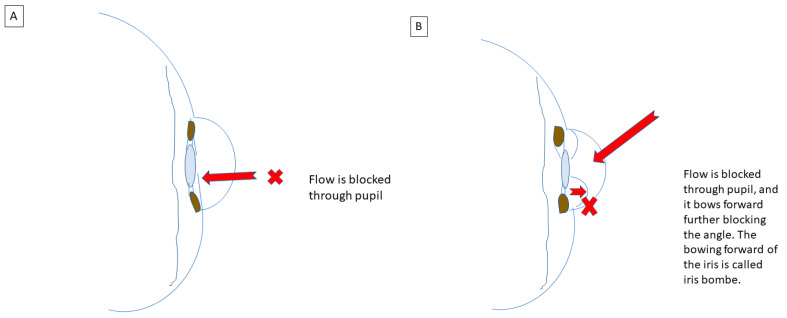
Sketch of (**A**) angle closure glaucoma due to blockage of flow through the pupil from the posterior chamber to the anterior chamber. (**B**) Illustration of iris bombe, which occurs when flow is blocked through the pupil and the iris bows forward, further blocking the angle. (Schematic by M.D.).

In closed-angle glaucoma, access to the outflow pathways is physically blocked. The most common mechanism of angle closure is pupillary block. This occurs when the central iris (adjacent to the pupil) contacts the anterior surface of the lens, blocking aqueous flow from passing through the pupil to the anterior chamber (see Figure 4A). The subsequent accumulation of aqueous humor pushes the peripheral iris anteriorly into the trabecular meshwork, a phenomenon known as iris bombe, closing off the angle and blocking outflow drainage, with a rise in IOP (see Figure 4B). These mechanisms, when in place chronically, can result in sustained outflow obstructions and facilitate the formation of adhesions between the iris, lens, and drainage angle. The adhesions that form between the lens and the iris are called posterior synechiae (PS) and the adhesions that form between the iris and the drainage angle are called peripheral anterior synechiae (PAS). Unlike most other types of glaucoma, angle closure glaucoma in the acute setting can cause blurred vision, eye pain, headache, and nausea from the elevated IOP. Secondary mechanisms resulting in angle closure include ischemia and inflammation. Ischemia leads to neovascularization (abnormal growth of new vessels) in the anterior chamber angle where the associated fibrovascular membranes from the abnormal vessels contract and grow onto the angle, effectively closing off the angle. Another mechanism is abnormal accumulation of fluid or mass effect behind the iris and lens that can push the lens and iris forward, blocking off the angle and leading to elevated IOP. Inflammation, also described later, results in the formation of adhesions (PS and PAS) that affect aqueous outflow.

### 1.5. Mechanisms Leading to Optic Nerve Damage

The elevation in IOP, whether in open or closed-angle glaucoma, is thought to lead to optic nerve insult. How this elevation causes optic neuropathy is not yet fully understood, but there are two leading hypotheses: (1) mechanical and (2) vascular and autonomic dysfunction.

#### 1.5.1. Mechanical Dysfunction 

An elevation in IOP can lead to increased mechanical stress and strain on the lamina cribrosa and the adjacent tissues in the posterior pole of the eye. The bundles of RGC axons (fascicles) and soft tissue condense to form the optic nerve and traverse the lamina cribrosa before exiting into the optic canal. This area where the sclera meets the lamina cribrosa can be particularly sensitive to elevated IOP [5]. An elevation in pressure can cause increased stress and strain on these structures, leading to compression, deformation, or remodeling of the lamina cribrosa. This can cause subsequent mechanical damage to the RGC axons and disturb axonal transport. Downstream effects include possible mitochondrial dysfunction in RGCs and astrocytes, thus making it difficult to meet the increased energy demands, i.e., IOP-induced metabolic stress.

#### 1.5.2. Vascular Dysfunction

Ischemia and local disruption to vascular autoregulation can also disrupt metabolic function in the RGC and its axons. It is thought that risk factors including increased IOP, vascular bed abnormalities, endothelial cell damage, and atherosclerosis disrupt and reduce ocular blood flow. This in turn causes ischemia and damage to the connective tissues and axons of the optic nerve head. Studies have demonstrated that patients with primary open-angle glaucoma exhibit ocular vascular dysregulation in the choroid, optic nerve head, central retinal artery, and perifoveal macular capillaries [6]. The dysregulation has also been shown to extend beyond the cerebral vasculature [7]. Patients with open-angle glaucoma have impaired endothelial cell function including decreased responses to nitric oxide, vasoconstrictor endothelin-1, and vascular endothelial growth factor (VEGF) signaling pathways. Endothelial cell dysfunction and damage can affect the blood vessel diameter and increase resistance, leading to ischemia to distal tissues.

#### 1.5.3. Autonomic Dysfunction

The pathophysiology of open-angle glaucoma has also been associated with autonomic dysfunction. The autonomic nervous system is responsible for controlling the parasympathetic, sympathetic, and enteric regulation of multiple bodily functions. It has been hypothesized that the level of IOP may be partially controlled by the autonomic nervous system. Chemical stimulation to the hypothalamic regions that house central autonomic regulatory neurons have been shown to markedly increase IOP [8]. Moreover, patients with open-angle glaucoma have been observed to have higher variability in IOP throughout the day compared to healthy controls [9]. It has been hypothesized that the wider fluctuation in diurnal IOP and blood flow dysregulation contribute to the etiology of open-angle glaucoma, regardless of IOP level. Open-angle glaucoma patients have also demonstrated greater beat-to-beat variability in their heart rate compared to normal controls, another representation of the autonomic dysfunction associated with the disease [6].

### 1.6. Outline of the Review

Now that a better understanding of the categories of glaucoma and the mechanisms involved has been established, we want to move on to the discussion of this review. We outline the various systemic conditions that have associations with and/or can lead to the development of glaucoma. We will review these conditions by body system but recognize that there may be some overlap in certain areas. We would like note that we are not covering pregnancy and glaucoma in this review. However, we do advise that patients notify their providers and their obstetrics and gynecology team so that their treatment plans can be modified as needed during pregnancy. Among the systems we will cover are pharmacologically induced glaucoma; inflammatory and auto-immune conditions; infectious diseases; dermatologic, cardiovascular, pulmonary, renal, urologic, neurologic, psychiatric, systemic malignancies; intraocular tumors; as well as pediatric and genetic conditions. Hopefully, with the help of this review, there may be a better understanding of how systemic conditions and their respective treatments may predispose a patient to glaucoma.

## 2. Pharmacologically Induced Glaucoma

### 2.1. Medications Associated with Glaucoma 

Certain classes of medications, including sympathomimetics, anticholinergics, antihistamines, antidepressants, and sulfa-based drugs that are prescribed for the treatment of systemic diseases including bladder, pulmonary, allergic, psychiatric, and neurologic conditions, have been shown to cause episodes of acute angle closure glaucoma by pupillary block and non-pupillary block. The risk of pupillary block is particularly increased in patients with pre-existing anatomically narrow drainage angles. In non-pupillary block angle closure glaucoma, patients with anomalous irides can have thicker tissue at the peripheral iris which crowds the iridocorneal angle as it dilates, increasing the resistance to aqueous flow. Another mechanism in non-pupillary block is anterior movement of the ciliary body toward the peripheral iris from choroidal effusion as an idiosyncratic drug reaction [10,11]. Medications that can cause idiosyncratic reactions include topiramate and sulfa drugs. While the mechanisms of each medication causing acute angle closure may vary and can occur in patients with anatomically normal angles, patients with anatomically narrow angles have a higher risk when taking the medications listed in Table 1 [12,13].

#### 2.1.1. Topiramate

Topiramate, brand name Topamax, is an anti-seizure medication that is commonly prescribed for migraine and seizure prophylaxis, alcohol use disorder, obesity, and psychiatric disorders. While the drug can be very effective for the aforementioned illnesses, it can cause a rare side effect of bilateral angle-closure glaucoma via the development of ciliochoroidal effusions. These effusions can cause anterior rotation of the ciliary body and anterior displacement of the lens–iris diaphragm [14]. Combination medication of topiramate with phentermine is a commonly prescribed weight loss prescription that has also been reported to rarely cause angle-closure glaucoma through ciliochoroidal effusions [12].

#### 2.1.2. Anticholinergic and Cholinergic Agonists

Anticholinergic medications form a class of medications that have been well established as a cause of angle closure by pupillary block. Even medications that have partial anticholinergic properties like antihistamines have been reported to induce angle closure in patients with anatomically narrow angles. Interestingly, while topical cholinergic medications are often used to treat acute angle closure glaucoma, the use of some agents, such as pilocarpine, has also been reported to cause angle closure by forward displacement of the lens–iris diaphragm [10]. It should be noted that the use of pilocarpine is contraindicated in patient with topiramate-induced angle closure, as the ciliary body may be further triggered with the use of pilocarpine [14].

### 2.2. Association of Corticosteroid Use with Glaucoma

Glucocorticoids can increase intraocular pressure, particularly in patients who use ophthalmic glucocorticoid drops. This response has also been observed in patients on both long- and short-term topical drops as well as inhaled, oral, and intravenous systemic steroids for conditions such as chronic obstructive pulmonary disease (COPD) and rheumatologic conditions. This response is more pronounced in patients with primary open-angle glaucoma.

Risk factors that increase the risk of corticosteroid-associated glaucoma include patients with primary open-angle glaucoma, high myopia, eyes with a history of penetrating keratoplasty or refractive surgery, age < 10 years or older-age adults [15], DM, connective tissue disease (rheumatoid arthritis and COPD in particular), pigment dispersion syndrome, traumatic angle recession, and endogenous hypercortisolism. It should be noted that steroid-induced glaucoma after refractive surgery can be masked by falsely low IOP due to thin central corneal thickness, ocular rigidity changes, corneal edema, and fluid accumulation beneath the LASIK flap [16].

The elevation in intraocular pressure due to corticosteroid administration is dependent on dose, chemical structure, frequency of administration, route of administration, duration of treatment, and each patient’s individual susceptibility to steroid responses. Although a “steroid response” can occur at any time after the initiation of a therapy, it is most often seen 2–6 weeks after initiation. Patients more at risk for steroid-induced glaucoma are children under the age of 6 years and the elderly [17]. Approximately 4–6% of the population are considered “high responders” to steroids, which develop an IOP greater than 31 mm Hg or an increase more than 15 mm Hg from their baseline. One-third of the population are considered to be “moderate responders”, and they develop an IOP between 25–31 mm Hg or an increase of 6–15 mm Hg from their baseline. The rest of the population are considered non-responders. The characteristics of steroid responders are outlined in Table 2. Clinicians should also note that the response can vary depending on the potency of steroid [17].

#### 2.2.1. Mechanisms of Corticosteroid-Induced IOP Elevation

Corticosteroids have been studied and reported to raise IOP by increasing resistance to aqueous humor outflow by inducing biochemical and morphological changes in the trabecular meshwork. There are several hypotheses for explaining this mechanism. One hypothesis is that steroids lead to an accumulation of polymerized glycosamino-glycans (GAGs) in the trabecular meshwork by stabilizing their lysosomal membranes. The polymerized GAGs become hydrated and act to increase outflow resistance. Steroids are also hypothesized to increase the expression of extracellular matrix proteins like fibronectin, GAGs, elastin, and laminin within the trabecular meshwork, further increasing aqueous humor outflow resistance.

#### 2.2.2. Management of Corticosteroid-Induced IOP Elevation

Patients receiving topical steroid therapy should be evaluated for IOP checks by their providers two weeks after the initiation of therapy. They should receive follow-up IOP checks every 4 weeks after the initial visit for 2–3 months, then every 6 months if steroid therapy continues [18]. Patients who receive ocular steroid injections should be monitored for IOP elevation for several months, as many studies have shown delayed IOP elevations [18]. In those receiving systemic steroids, IOP should be checked at 1, 3, and 6 months, and every 6 months after for the duration of treatment [18]. The risks associated with different routes of steroid administration are outlined in Table 3.

### 2.3. Management of Drug-Induced Glaucoma

Patients who are prescribed corticosteroids should be monitored prior to and after steroid use if the medication will be administered for more than a few weeks. For steroid-induced open-angle glaucoma, lower-potency alternatives or non-steroidal anti-inflammatory medications (i.e., diclofenac or ketorolac) should also be considered. Any patients who are taking the medications including, but not limited to those listed in Table 1 should be educated on the signs and symptoms associated with angle-closure and that they should be advised to see an ophthalmologist urgently if they experience these symptoms. For the medications listed below, the management is discontinuation of the offending agent, along with topical and systemic IOP-lowering medications. For topiramate or sulfa-drug-induced angle-closure, adding cycloplegic agents (i.e., atropine or cyclopentolate) and oral steroids to accelerate resolution of the choroidal effusions should be considered. Cycloplegic agents paralyze the ciliary body, allowing the lens–iris diaphragm to shift posteriorly to open the angle. Systemic steroids may also be considered in severe or refractory cases. A recent report suggests that Rho Kinase inhibitors can also be effective at treating steroid-induced glaucoma [26].

## 3. Inflammatory/Autoimmune and Infectious Diseases

Inflammatory or infectious diseases of the eye can manifest in the eye as uveitis. Uveitis is ocular inflammation stemming from the inflammation of uveal tissues (iris, ciliary body, and choroid). There can be subsequent uveitic glaucoma from elevated IOP (which occurs via obstruction of the trabecular meshwork due to accumulation of inflammatory particles, swelling of the trabecular lamellae, loss or damage to the endothelial cells of the trabecula, and/or direct injury to the trabecula) in addition to synechiae (from PS and PAS formation), as noted in the introduction. Systemic inflammatory causes of uveitic glaucoma include Human Leukocyte Antigen B27 (HLA-B27) related diseases such as ankylosing spondylitis, psoriasis, inflammatory bowel disease, and reactive arthritis [27]. It is estimated that 50% of cases of acute anterior uveitis are associated with the HLA-B27 allele [28]. Other systemic inflammatory conditions known to cause uveitis include juvenile idiopathic arthritis and sarcoidosis [29]. Approximately 20% of patients in the United States with uveitis develop glaucoma [30]. A list of systemic inflammatory and infectious causes of glaucoma are listed in Table 4.

Patients with uveitis have an increased risk of developing glaucoma not only from the inflammatory nature of uveitis itself but from the use of corticosteroids for the treatment of uveitis, in up to one third of patients. The pathogenesis and risk factors associated with steroid-induced glaucoma are discussed in the section Pharmacologically Induced Glaucoma. A rarer form of angle-closure glaucoma can result from Vogt–Koyanagi–Harada syndrome, in which intraocular inflammation and edema can result in choroidal effusions and cause the ciliary body to rotate forward and close off the anterior chamber drainage angle [31]. The inflammatory cells can subsequently release cytokines that further worsen the inflammation and potentially lead to neovascularization of the drainage angle.

### Management of Intraocular Inflammation

First line management of intraocular inflammation or infection is to treat the underlying cause. Patients should be treated with the appropriate antimicrobial medications, steroids or NSAIDs, immunosuppressive medication, and/or biologic agents. Elevated IOP is addressed medically with aqueous suppressants or surgery with surgical iridectomy, filtration, drainage devices, or cyclo-destructive procedures if elevated pressure does not respond to the above treatments. Patients can require local therapy with topical steroids or localized injections of steroids either sub-Tenon or intravitreal. The use of topical corticosteroids should be used judiciously and IOP should be monitored for steroid-induced elevations.

## 4. Dermatologic Conditions Associated with Glaucoma

### 4.1. Vitiligo

Vitiligo is an autoimmune disorder characterized by melanocytic destruction and depigmented maculae. Patients with this condition may have ocular pigmentary abnormalities in the retina and iris. Studies have found associations with vitiligo and increased risk of developing normal-tension glaucoma, suggesting prompt ophthalmic consultation and close follow-up [32].

### 4.2. Cutaneous Sarcoidosis

Dermatologic manifestations of cutaneous sarcoidosis can include papules, plaques, subcutaneous nodules, lupus pernio, infiltration of scarring, alopecia, and ulcerative lesions. While dermatologic manifestations are usually a sign of chronic sarcoidosis, there are no correlations between skin findings and the degree of systemic involvement in sarcoidosis [33]. Ocular sarcoidosis classically presents as anterior uveitis which can lead to uveitic glaucoma, discussed in more detail in the section Inflammatory/Autoimmune and Infectious Diseases. Patients with sarcoidosis should receive frequent ocular examinations even in the absence of symptoms. The mainstay of treatment for sarcoidosis consists of systemic corticosteroids.

### 4.3. Cutaneous Manifestations from Systemic Inflammatory Diseases

Many systemic inflammatory diseases have cutaneous manifestations, including rheumatoid arthritis, systemic lupus erythematosus, scleroderma, and systemic vasculitis syndromes. These conditions can have ocular involvement including retinal vasculitis, uveitis, and scleritis. Patients with systemic inflammatory conditions should receive careful ocular examinations for potential vision-threatening complications such as glaucoma. These conditions are discussed in more detail in the section Inflammatory/Autoimmune and Infectious Diseases.

## 5. Cardiovascular Conditions Associated with Glaucoma

### 5.1. Diabetes Mellitus and Diabetic Retinopathy

Diabetic retinopathy (DR) is a vision-threatening complication of uncontrolled DM in which there is growth of new anomalous vessels in the inner retina, known as neovascularization, in response to chronic microvascular ischemia from uncontrolled blood glucose. The vessels can extend into the vitreous and with sufficient ischemia, they can grow onto the iris and into the drainage angle in what is known as neovascular glaucoma (NVG). NVG is considered an ophthalmic emergency and requires immediate treatment due to the rapid increase in IOP with potential vision loss. NVG can occur insidiously and asymptomatically until the IOP suddenly rises after a sufficient portion of the drainage angle has been closed. The greatest risk factors for developing neovascular glaucoma are proliferative (DR) and central retinal vein occlusions (CRVO). DM is also a risk factor for developing CRVO, although this may be due to its association with other systemic factors that increase cardiovascular risk including hypertension and hyperlipidemia [34,35]. Of note, topical beta-blockers may have a rare side effect of inducing severe hypoglycemia in patients with insulin-dependent DM, necessitating careful monitoring of blood sugars in this population when started on topical beta-blockers [36].

### 5.2. Systemic Hypertension

Systemic hypertension can be associated with elevated IOP. This may not necessarily lead to glaucoma as the relative increase is modest. The relationship between elevated blood pressure and IOP is still being investigated. A systematic review and meta-analysis concluded that an increase in systolic blood pressure by 10 mm Hg was associated with an increase in intraocular pressure by 0.26 mm Hg. An increase of 5 mm Hg in diastolic blood pressure was associated with an increase in IOP by 0.17 mm Hg [37].

#### Hypertension and Central Retinal Vein Occlusion

The main mechanism through which hypertension is a risk factor for glaucoma is through the development of a CRVO. An ischemic CRVO can lead to NVG as the decreased oxygenation can progress to neovascularization and closure of the drainage angle in 18–60% of cases, which can develop anywhere from 2 weeks to 2 years after the inciting CRVO [38]. Up to 73% of patients with CRVO over the age of 50 and up to 25% of patients who develop CRVO under the age of 50 have a history of systemic hypertension [39]. A third of patients with CRVO may also have systemic hyperlipidemia [39]. It should be noted that taking oral contraceptive pills is the most common underlying association for young patients to develop a CRVO and that it should be stopped immediately if a CRVO occurs.

Patients with evidence of cardiovascular disease (CVD) should be counseled about the risk for developing ocular complications and emphasize the importance of blood pressure, lipid, and blood sugar management [38].

### 5.3. Hypotension

The effects of hypotension appear to be more damaging to the optic nerve in comparison to hypertensive effects. Low blood pressure has been well established to be associated with an increased risk for glaucoma. The hypothesized mechanism is that hypotension can lead to ischemic injury. This occurs as drops in blood pressure cause a decrease in ocular perfusion pressure, calculated as arterial blood pressure minus intraocular pressure, both of which have been shown to be risk factors for glaucoma [40]. Ocular perfusion pressure relies on autoregulation to consistently supply enough blood flow to match the metabolic requirements of ocular tissues like oxygen, nutrients, and removal of waste products. In the Early Manifest Glaucoma Trial, lower systolic blood pressure in patients with lower IOP at baseline was associated with faster primary open-angle glaucoma disease progression [41]. In the Thessaloniki Eye Study, low diastolic ocular perfusion pressure was associated with an increased risk for primary open-angle glaucoma in subjects using antihypertensive treatment [42].

Moreover, nocturnal dips in blood pressure and their relation to glaucoma progression have been explored in numerous studies. Physiologically normal dips of systolic BP by 10–20% occur due to a decrease in sympathetic nervous system activity during sleep. While physiological dips provide cardiovascular protection [43], nocturnal dips in blood pressure greater than 10% have been associated with increased visual field loss in patients with glaucoma [44].

### 5.4. Carotid-Cavernous (CC) Fistula

A carotid-cavernous fistula causes abnormal communication between the cavernous sinus and carotid artery. This causes arterial flow and venous engorgement leading to elevated episcleral venous pressure. Aqueous humor outflow is dependent on the pressure gradient between IOP and episcleral venous pressure. Aqueous humor drains through the trabecular meshwork into the canal of Schlemm, which then drains into aqueous and episcleral veins. The pressure at which the aqueous humor meets the episcleral veins is called the episcleral venous pressure, which is a retrograde pressure that slows down the rate of aqueous humor drainage. Elevated episcleral venous pressures can decrease the rate of aqueous humor drainage, and cause elevations in IOP. The episcleral veins then drain to the superior and inferior ophthalmic veins to the cavernous sinus, where it rejoins the systemic circulation. Thus, disease entities that disrupt the flow of aqueous humor into the systemic circulation can increase episcleral venous pressure. This also causes a dilation of retinal veins and optic disc swelling that can concurrently damage optic nerve fibers.

### 5.5. Pseudoexfoliation Syndrome

Pseudoexfoliation syndrome is an age-related systemic disease where protein-like fibrillar material deposits in many extraocular organs (heart, lungs, kidneys, liver, skin, and blood vessels), but most commonly deposits intraocularly in the anterior segment of the eye (pseudoexfoliative material) in the anterior lens capsule [45]. Pseudoexfoliative syndrome has strong associations with a risk for developing secondary open-angle glaucoma, as up to 50% of patients with pseudoexfoliation syndrome develop pseudoexfoliation glaucoma [45]. It is the most common form of secondary open-angle glaucoma. In the Blue Mountain Eye Study, patients with pseudoexfoliation syndrome had up to a three-fold higher risk of developing glaucoma, while those with pseudoexfoliation syndrome in the eye had up to a five-fold increased risk [45]. The flaky material peels off the outer lens capsule and collects in the angle, directly clogging the trabecular meshwork, which leads to increased eye pressure. The relationship between pseudoexfoliation syndrome and homocysteine is discussed in the section Alzheimer’s Disease, Pseudoexfoliation Syndrome, and Glaucoma.

## 6. Pulmonary Conditions Associated with Glaucoma

### 6.1. COPD and Obstructive Lung Patterns

While the literature behind chronic obstructive pulmonary disease (COPD) and its associations with glaucoma are scarce, a study from Korea found that patients with COPD have an increased risk of developing open-angle glaucoma compared to people who had normal or restrictive lung patterns [46]. This risk is even higher in non-smoking women. Studies suggest that COPD causes oxidative stress and hemodynamic abnormalities, mechanisms that have been associated with the pathogenesis of glaucoma. The use of inhaled or oral corticosteroid, ipratropium, and anticholinergics in the management of COPD, particularly in chronic cases, is also associated with developing glaucoma and is discussed further in the section Pharmacologically Induced Glaucoma. Of note, the use of topical beta blockers, particularly non-selective ones like timolol, levobunolol, and metipranolol, can in rare cases exacerbate COPD and other obstructive lung diseases. Thus, patients with a medical history of obstructive lung disease should be carefully monitored for this potential adverse reaction if started on these medications [47].

### 6.2. Asthma

While asthma does not directly cause nor increase the risk for developing glaucoma, the use of inhaled corticosteroids could cause elevation in IOP by increased resistance within the trabecular meshwork (explained further in Pharmacologically Induced Glaucoma) and may need to be observed more closely if the patient exhibits other risk factors for glaucoma such as family history and increased cupping of the optic nerve.

### 6.3. Obstructive Sleep Apnea

Obstructive sleep apnea-hypopnea syndrome is a disease characterized by recurrent episodes of complete or partial upper airway collapse during sleep. Patients may also experience daytime sleepiness, fatigue, and cognitive impairment. The literature shows evidence that patients with OSA have a higher prevalence of glaucoma, particularly in patients with severe disease with apnea-hypopnea index > 30 [48]. Patients with glaucoma, particularly NTG, also have a higher risk of developing sleep disorders. Many patients with OSA have been found to have changes in intraocular pressure, retinal nerve fiber layer thinning, and alteration of the visual field with no previous history or evidence of glaucoma or glaucomatous changes on ophthalmic examination. Vascular and mechanical explanations for the optic nerve involvement in OSA have been proposed. Vascular reasons include recurrent hypoxia, elevated vascular resistance, oxidative stress, and decreased cerebral perfusion pressure that can cause hypoxic damage to the optic nerve. Mechanical factors include spikes in IOP at night due to being in a supine position while asleep, obesity, and elevated intracranial pressure. Management of patients with OSA includes ophthalmic evaluations, and patients with glaucoma should be evaluated for sleep disorders, particularly those with normal-pressure glaucoma. Patients with evidence of OSA or glaucomatous damage despite normal IOP can benefit from continuous positive airway pressure [49].

## 7. Renal Conditions Associated with Glaucoma

### Chronic Kidney Disease

Patients with chronic kidney disease (CKD) have been found to have an increased risk of developing ocular diseases including glaucoma, DR, age-macular degeneration, and cataracts compared to patients who did not have CKD [50,51,52]. The association between CKD and glaucoma, however, has not been elucidated until a recent meta-analysis of 12 studies found that those with CKD had a significantly higher odds ratio of developing glaucoma compared to those without CKD [51]. They found that the risk for developing glaucoma in patients with CKD increases with chronicity of CKD, East Asian ancestry, diabetes mellitus, cigarette smoking, and primary open-angle glaucoma [48,49,50]. While the pathophysiology is not entirely clear, CKD and glaucoma share many mechanistic similarities including oxidative stress, renin-angiotensin system dysfunction, and atherosclerosis that lead to hypoperfusion of the optic nerve [51]. It is also thought that the hypoperfusion can consequently lead to reperfusion injury, resulting in glaucomatous damage to the RGCs. Although this case is extremely rare, the report highlights the importance of understanding each patient’s comorbid conditions to monitor side effects and adverse interactions of treatment. The association between CKD and glaucoma emphasizes the importance of referring patients with risk factors that can exacerbate CKD including diabetes, hypertension, and cardiovascular disease for routine eye exams and IOP checks as a preventative health strategy for potentially irreversible glaucomatous damage. Moreover, management of the aforementioned diseases with control of blood pressure, blood sugar, and cholesterol can further prevent the wide-reaching effects of both CKD and glaucoma.

Although rare, there have been reports of adverse reactions including metabolic acidosis and anemia following systemic absorption of the topical carbonic anhydrase inhibitor dorzolamide, prescribed for the treatment of glaucoma in patients with CKD [53].

## 8. Urologic Conditions Associated with Glaucoma

### Overactive Bladder

Oral anticholinergic therapy is a commonly prescribed group of medications for the treatment of overactive bladder and urinary urge incontinence. These medications work by blocking the M3 muscarinic receptors located on the detrusor muscle in the urinary bladder to prevent unwanted contraction of the smooth muscles [54]. Anticholinergic medications such as ipratropium bromide, atropine, and oxybutynin can cause a mydriatic effect and subsequent pupil dilation, potentially precipitating angle closure glaucoma in patients that have pre-existing anatomically narrow angles [54]. Although angle closure is a very rare complication, patients with a history of glaucoma or anatomically narrow angles should consult with their ophthalmologist when prescribed anticholinergics for urologic conditions.

## 9. Neurologic Conditions Associated with Glaucoma

### 9.1. Alzheimer’s and Other Neurodegenerative Diseases

There have been many studies in recent years exploring the relationship between glaucoma and chronic neurodegenerative processes like Alzheimer’s disease (AD) and Parkinson’s disease. The association between glaucoma and AD has been a topic of study because they share several common features, including the age-related and irreversible loss of selective types of neurons. Epidemiologic studies have shown an increased prevalence of glaucoma in patients with AD compared to non-AD [55,56]. MRI studies have shown trans-synaptic degeneration affecting the central areas of the visual system in glaucomatous patients [57], suggesting that glaucoma may involve a more complex neurodegenerative process that affects the entire visual system, extending beyond purely ocular involvement.

#### 9.1.1. Mechanistic Associations between Glaucoma and Alzheimer’s Disease

In the laboratory, human ocular tissue samples from patients with glaucoma who had undergone operations were compared to normal tissues. In glaucomatous patients, there were decreased concentrations of normal Tau protein and increased concentrations of abnormally hyperphosphorylated Tau protein [58]. This supports the association between glaucoma and AD because there is elevated abnormally hyperphosphorylated tau protein in the CSF of AD patients [58]. Another study detected lower levels of amyloid beta proteins and increased levels of tau proteins in the vitreous fluid, consistent with the pattern found in the CSF of patients with AD [59]. The mechanism is hypothesized to involve the RGC axons that normally transport amyloid precursor protein to distal synaptic targets. Thus, disruption or damage to the RGC axon transport of amyloid precursor may lead to elevated levels of amyloid-B detected in the posterior retina at the outer border of the inner nuclear layer and aqueous humor [58]. Glaucoma is sometimes referred to as “ocular Alzheimer’s disease” due to its similar pathogenetic mechanisms.

#### 9.1.2. Alzheimer’s Disease, Pseudoexfoliation Syndrome, and Glaucoma

Interestingly, Alzheimer’s disease and pseudoexfoliation syndrome (discussed previously in Cardiovascular Conditions Associated with Glaucoma), both of which are risk factors for developing glaucoma, have a higher prevalence of elevated homocysteine levels [60]. Pseudoexfoliation syndrome is an age-related systemic disease that involves the accumulation of fibrils containing amyloid-related proteins particularly in the anterior lens capsule, which may have similarities to the pathophysiology behind Alzheimer’s disease.

The ongoing research to find associations between glaucoma and neurodegenerative diseases in its pathophysiology will hopefully shed some new light to improve diagnosis and treatment of these diseases.

### 9.2. Migraines

Migraine is a chronic neurologic condition that is characterized by unilateral headache attacks associated with dysfunction of the autonomic nervous system [61]. Patients with migraines have an increased risk of developing vascular comorbidities and were found to have a higher incidence of open-angle glaucoma compared to individuals without migraines. However, it is currently unknown if migraines are indeed a risk factor for developing glaucoma or more simply a concomitant condition in patients with glaucoma. Moreover, the medications used to treat migraines can lead to medication-induced glaucoma. Topiramate is a commonly prescribed antiepileptic and migraine-prophylactic medication that can lead to angle closure. These medications are discussed in more detail in the section Pharmacologically Induced Glaucoma.

## 10. Psychiatric Conditions Associated with Glaucoma

While mood disorders, such as major depressive disorder, dysthymia, bipolar disorder, anxiety, and mood disorders due to medical conditions have not been implicated as being associated with glaucoma, the treatment regimen for these disorders can lead to medication-induced angle-closure glaucoma. Antidepressants, like venlafaxine and escitalopram, and antipsychotics, like aripiprazole, can cause pupillary block. Some medications, particularly sulfa-related drugs, can also cause angle closure by inducing ciliary effusions. It should be noted that patients with glaucoma were found to have a higher prevalence of depression compared to those who did not have glaucoma [62]. In these cases, recognition and removal of the inciting agent is critical to resolving the angle closure and requires prompt evaluation by an ophthalmologist.

## 11. Systemic Malignancies and Intraocular Tumors

### 11.1. Association of Intraocular Tumors and Elevated IOP

Intraocular tumors can lead to elevations in IOP and damage the structures within the globe. This is usually due to multiple mechanisms ranging from direct seeding of the angle with tumor cells, mass effect of the tumor which physically closes the drainage angle, secondary inflammation/bleeding from the tumor, and/or other secondary effects such as autoimmune response or ischemic effect leading to neovascularization of the anterior chamber drainage angle resulting in a secondary angle closure glaucoma. Patients who present with unilateral or very asymmetric glaucoma should be considered for possible malignancy. The characteristics of the tumor including size, location, tissue type, and degree of inflammation, necrosis, and hemorrhage can affect the severity of IOP elevation [63].

### 11.2. Metastasis to the Eye from Systemic Malignancies

Metastasis from systemic malignancies to the eye can also cause secondary glaucoma. The choroid is the most common ocular structure for metastasis from systemic malignancy, but secondary glaucoma is most likely to result from metastasis to the anterior segment [63]. The rate of developing secondary glaucoma from metastasis to the choroid is 5–7.5% and increases to 56–67% from metastasis to the anterior segment [63,64]. Invasion of the anterior chamber angle and trabecular meshwork are the most common mechanisms for obstructing aqueous humor outflow and causing elevated IOP. The differential diagnosis for metastasis from systemic malignancies is listed in Table 5.

### 11.3. Primary Intraocular Tumors

Primary intraocular malignancies have also been identified as a cause of secondary glaucoma. Among these, uveal melanomas are the most common. As discussed, the location of the melanoma can determine the risk and severity of the associated secondary glaucoma. Melanomas in the ciliary body are at the highest risk, followed by the iris and choroid. Choroidal melanomas elevate the IOP by causing neovascularization of the iris and synechial angle closure [64]. Larger tumors in the posterior eye can cause angle closure as complications such as retinal detachment or choroidal hemorrhage can induce anterior displacement of the lens–iris diaphragm that secondarily obstructs aqueous outflow. Iris and ciliary body melanomas increase IOP by directly invading the angle, causing dysfunction to aqueous drainage. Other primary intraocular malignancies implicated in the development of secondary glaucoma are listed in Table 6.

## 12. Pediatric and Genetic Diseases

### 12.1. Introduction to Glaucoma in the Pediatric Population

The incidence of glaucoma in the pediatric population (age < 20 years) is 2.29 per 100,000 patients in the United States [65]. Primary congenital glaucoma has a prevalence of approximately 2.85 per 100,000 patients [65] The majority of cases of pediatric patients with eye malformations are associated with genetic syndromes, while about 21% are isolated [66]. Genetic causes of early-onset glaucoma are rare, including juvenile open-angle glaucoma, anterior segment dysgenesis, congenital glaucoma, and familial normal-tension glaucoma [67]. Glaucoma associated with the systemic syndromes listed in Table 7 often present in childhood. Although the occurrence of childhood glaucoma is rare, we think it is important for providers who manage the pediatric population to be aware of potential ocular complications like glaucoma of patients who present with a constellation of symptoms consistent with genetic syndromes.

The mechanisms behind childhood glaucoma associated with systemic diseases are often separated into neural crest, phakomatoses, and metabolic abnormalities. Eye manifestations of neural crest disorders include abnormal anatomical development of the anterior chamber, predisposing patients to an increased risk of developing secondary glaucoma due to decreased aqueous humor drainage. In conditions such as Marfan Syndrome, patients are at risk for lens subluxation because the intraocular lens is prone to malformation and can displace anteriorly, resulting in pupillary block [73].

Phakomatoses (sometimes known as neurocutaneous syndromes) are a group of congenital disorders typically involving the skin as well as the central and peripheral nervous systems, and can lead to glaucoma by various mechanisms. The most common syndromes associated with glaucoma are neurofibromatosis (von Recklinghausen), Sturge–Weber, and Von Hippel–Lindau disease. While rare, secondary glaucoma can occur with neurofibromatosis I due to infiltration of the anterior chamber by neurofibromatosis tissue, abnormal development of the drainage angle, and/or growth of Lisch nodules in the drainage angle leading to elevated eye pressure [73]. In Sturge–Weber, glaucoma occurs from abnormal development of the drainage angle and increased episcleral venous pressure [73]. Von Hippel-Lindau disease can lead to glaucoma via neovascularization and NVG, secondary to formation of retinal capillary hemangiomas [73].

Metabolic disorders have also been associated with the development of secondary glaucoma, typically through anomalous development of the anterior chamber and deposition of extracellular material in the drainage angle and the trabecular meshwork [73]. One such example is homocystinuria, a disorder in which anterior dislocation of the intraocular lens can cause acute pupillary block glaucoma.

Patients with these genetic syndromes can often have different presenting symptoms, requiring a multidisciplinary care team. It is therefore imperative that these care teams be aware of the ocular and other systemic effects of these diseases and involve eye and other specialists to prevent, diagnose, and treat these patients before sight- or life-threatening complications arise.

### 12.2. Management of Pediatric Glaucoma

Patients with glaucoma associated with pediatric syndromes often do not respond to medical glaucoma treatments as effectively as adults, as they can be complicated by abnormal development of the drainage angle. They sometimes require surgery to manually open the drainage angle to bypass the maldeveloped trabecular meshwork. Pediatric patients presenting with the triad of symptoms of photophobia, epiphora, or blepharospasm need to be evaluated urgently by an eye specialist as this could be an indication of congenital glaucoma.

## 13. Conclusions

Glaucoma is a group of diseases characterized as a degenerative optic neuropathy with cupping of the optic nerve head and visual field loss that is a leading cause of blindness in the US and worldwide. Open- and closed-angle glaucoma can occur as primary and secondary diseases. In this review, we discussed the categories of glaucoma, the mechanisms involved, risk factors, and the secondary causes of glaucoma. We outlined the various systemic conditions that have associations with and/or can lead to development of glaucoma by body system, including systemic malignancies and intraocular tumors; neurologic, pulmonary, renal, cardiovascular, urologic, dermatologic, auto-immune conditions; infectious conditions; pediatric and genetic conditions; and pharmacologic causes. We hope that our discussion of systemic conditions, their commonality or rarity, mechanisms, treatments, and their associations with developing glaucoma emphasizes the importance of ocular examinations and follow-up with the multidisciplinary teams involved in the care of each patient to prevent unnecessary vision-loss.

## Figures and Tables

**Table 1:** Medications associated with glaucoma risk.

Medications Associated with Risk of Glaucoma
Systemic sympathomimetics
Epinephrine Ephedrine Pseudoephedrine Dipivefrin Apraclonidine Salbutamol
Anticholinergic
Tiotropium bromide Tropicamide Atropine Ipratropium bromide
Cholinergic
Pilocarpine
Antihistamines
Cimetidine Ranitidine Orphenadrine
Depression/Anxiety Medications
Amitriptyline Imipramine Mianserin hydrochloride Paroxetine Fluoxetine Maprotiline Fluvoxamine Venlafaxine Citalopram Escitalopram Duloxetine
Sulfa-Based Drugs
Acetazolamide Hydrochlorothiazide Cotrimoxazole Topiramate
Others
Sumatriptan Lactulose Metoclopramide

**Table 2:** Characteristics of steroid responders.

Type of Responders	Prevalence	Definition
High	4–6% of the Population	IOP > 31 mm Hg or Increase in IOP > 15 mm from baseline
Moderate	⅓ of the population	IOP between 25–31 mm Hg or Increase 6–15 mm Hg from baseline
Non-Responders	⅔ of the population	IOP < 20 mm Hg or Increase < 6 mm from baseline

**Table 3:** Risk of developing elevated IOP based on method of steroid administration.

Method of Steroid Administration	Risk of Developing Increased IOP > 20 mm Hg
Topical Ocular	Up to 3% in patients taking Difluprednate [19]
Periocular (subconjunctival, retrobulbar, sub-tenon)	Up to 7.4% [20]
Intravitreal	Up to 33–66% [21,22]
Systemic—Oral	Odds ratio of up to 1.41 [23]. Risk is correlated with dosage of corticosteroid
Systemic—Inhaled	No increased risk reported in patients without a family or prior history of glaucoma [24]. Odds ratio up to 2.6 in patients with a family history of glaucoma [25].

**Table 4:** Differential Diagnosis for Inflammatory and Infectious Causes of Glaucoma.

Inflammatory and Infectious Conditions
Acute retinal necrosis
Behcet disease
HLA B27—related acute anterior uveitis
Juvenile idiopathic arthritis-associated uveitis
Sarcoidosis
CMV retinitis
Congenital rubella
Disseminated meningococcemia
Hansen disease (leprosy)
Herpes virus-associated uveitis
Lyme disease
Syphilis
Toxocariasis

**Table 5:** Differential Diagnosis for Ocular Metastasis from Systemic Malignancies.

Ocular Metastasis from Systemic Malignancies
Cutaneous melanomas
Leukemia:
Acute lymphocytic leukemia Acute myelogenous leukemia
Lymphoma
Non-Hodgkin lymphoma of the CNS Non-Hodgkin non-CNS involving lymphoma
Multiple Myeloma

**Table 6:** Differential Diagnosis for Intraocular Tumors Causing Secondary Glaucoma.

Primary Intraocular Tumors
Uveal melanoma
Retinoblastoma
Medulloepithelioma
Iris melanocytoma
Juvenile xanthogranuloma

**Table 7:** Differential Diagnosis for Pediatric and Genetic Causes of Glaucoma and their Mechanisms.

Condition	Mechanism(s) for Development of Glaucoma
Trisomy 21	Congenital glaucoma
Trisomy 16–18: Edward Syndrome	Congenital glaucoma
Trisomy 13–15: Patau Syndrome	Congenital glaucoma
Turner Syndrome	Anterior segment abnormalities [68]
Cretinism (Juvenile hypothyroidism)	Deposition of mucopolysaccharides and hyaluronic acids in the trabecular meshwork [69]
Diamond–Blackfan Syndrome	Congenital glaucoma [70]
Fanconi Anemia	Risk for angle closure due to microphthalmia [70]
Ehlers–Danlos Syndrome	Defect in type III collagen
Fetal Alcohol Syndrome	Congenital glaucoma
Homocystinuria	Anterior displacement of the intraocular lens
Kartagener Syndrome	Ciliary dyskinesia
Kimmelstiel–Wilson Syndrome	Neovascularization
Klinefelter Syndrome	Rare cases of goniodysgenesis/abnormal development of the drainage angle [71]
Marfan Syndrome	Lens subluxation, anterior displacement of a maldeveloped intraocular lens
Wilms Aniridia	Abnormal development of the drainage angle
Neurofibromatosis (von Recklinghausen)	Infiltration of the angle with neurofibromatosis tissue, abnormal development of the drainage angle [72]
Pierre Robin Syndrome	Severe congenital myopia
Sturge–Weber Syndrome	Abnormal development of the drainage angle [72]
Von Hippel–Lindau Disease	Neovascularization, abnormal development of the drainage angle [72]

## Data Availability

No new data were created during this study.
